# Diastolic Function in Hypertensive Patients with Basal Septal Hypertrophy Assessed by Conventional Methods and Diastolic Strain Rate: A Single-Center Cross-Sectional Study

**DOI:** 10.3390/jcm15207823

**Published:** 2026-10-10

**Authors:** Vlatka Reskovic Luksic, Marijan Pasalic, Marija Brestovac, Petra Angebrandt Belosevic, Sandra Jaksic Jurinjak, Jadranka Separovic Hanzevacki

**Affiliations:** 1 Department of Cardiovascular Diseases, University Hospital Centre Zagreb, 10000 Zagreb, Croatia; marijan.pasalic@yahoo.com (M.P.); marija.brestovac@gmail.com (M.B.); petraang37@yahoo.com (P.A.B.); sjaksicj@gmail.com (S.J.J.); jshanzevacki@gmail.com (J.S.H.); 2 Bioinformatics and Precision Medicine Research Group, Algebra Bernays University, 10000 Zagreb, Croatia; 3 School of Medicine, University of Zagreb, 10000 Zagreb, Croatia

**Keywords:** hypertension, echocardiography, speckle-tracking echocardiography, diastolic strain rate, diastolic dysfunction, basal septal hypertrophy

## Abstract

**Objectives**: To investigate early alterations in left ventricular (LV) diastolic function in young patients with uncomplicated arterial hypertension and basal septal hypertrophy (BSH), preserved LV ejection fraction (LVEF), and no overt cardiovascular comorbidities. **Methods**: Patients were divided into two groups according to the presence or absence of BSH and compared. Conventional diastolic assessment according to current recommendations were performed, along with two-dimensional speckle-tracking echocardiography for LV systolic and diastolic deformation and LA reservoir strain (LARS). **Results**: A total of 138 patients were included; 53.6% had BSH. For the whole population LV systolic and diastolic function were preserved, and overall LV geometry parameters and LA dimensions and function were within normal limits. However, patients with BSH were found to have more pronounced geometry changes in terms of concentric remodeling beyond BSH (LVMI 109.15 ± 25.33 vs. 89.19 ± 24.59 g/m^2^, *p* < 0.001; RWT 0.38 ± 0.11 vs. 0.3 ± 0.08, *p* < 0.001), LA dilation (32.68 ± 9.98 vs. 30.03 ± 11.37, *p* = 0.05), and impaired LV relaxation according to standard parameters (E wave 0.72 ± 0.18 vs. 0.79 ± 0.2 m/s, *p* = 0.05; A wave 0.76 ± 0.14 vs. 0.72 ± 0.19 m/s, *p* = 0.04; E/A 0.96 ± 0.28 vs. 1.16 ± 0.39, *p* = 0.001; e′ septal 0.08 ± 0.02 vs. 0.1 ± 0.02 m/s, *p* = 0.001; and e′ lateral 0.12 ± 0.03 vs. 0.15 ± 0.04 m/s, *p* = 0.001) and diastolic strain rate (SR) (SRe 1.17 ± 0.35 vs. 1.31 ± 0.35%/s, *p* = 0.02; SRe/SRa 1.39 ± 0.63 vs. 1.69 ± 0.89, *p* = 0.03). Measurement of the diastolic SR was feasible in 97.1% of the patients with good reproducibility and interobserver difference of 1.28%. Significant correlations were found between basal septal thickness and parameters of LV and LA remodeling and diastolic function. **Conclusions**: Basal septal hypertrophy appears to represent an early, functionally relevant stage of hypertensive myocardial remodeling rather than an isolated morphological finding. Even in the presence of preserved LVEF, BSH is associated with subtle impairment of LV relaxation. Deformation imaging may improve identification of patients at risk for progression toward overt diastolic dysfunction and heart failure with preserved ejection fraction.

## 1. Introduction

Chronic pressure overload in arterial hypertension (AH) induces structural adaptation of the left ventricle (LV), most commonly in the form of concentric hypertrophy. Regional remodeling, however, may precede global hypertrophy and often develops in the basal interventricular septum, leading to basal septal hypertrophy (BSH), which has been proposed as an early morphologic marker of hypertensive heart disease [1,2,3,4,5,6].

Left ventricular diastole is a complex, energy-dependent process comprising isovolumetric relaxation, early rapid filling, diastasis and atrial contraction. Because diastolic performance reflects the interplay between myocardial relaxation, restoring forces, chamber stiffness and filling pressures, no single echocardiographic parameter can comprehensively describe LV diastolic function [7,8]. Current recommendations therefore advocate a multiparametric approach [7,8]. Despite such algorithms, diastolic assessment frequently remains inconclusive, as each parameter is load-dependent and subject to methodological limitations.

Diastolic dysfunction represents an early manifestation of hypertensive heart disease [9,10,11,12,13,14,15,16,17]. Impaired active myocardial relaxation in hypertrophied myocardium may precede overt elevation of filling pressures, LA enlargement, and the development of heart failure with preserved ejection fraction (HFpEF) [10,12,13,14]. Even in uncomplicated hypertension, indices such as E/e′ and E/A ratio have demonstrated prognostic relevance [9,18,19,20]. Patients with BSH have been reported to exhibit higher filling pressures compared with healthy controls [21,22], suggesting that regional remodeling may carry functional consequences. Moreover, adequate treatment and control of blood pressure may improve diastolic LV function and prognosis [12,23], so it is important to recognize those in the early course of hypertensive heart disease [5].

Speckle-tracking echocardiography provides a sensitive assessment of myocardial mechanics and can detect subtle abnormalities even when conventional indices appear preserved [6,16,24]. In addition to LV systolic deformation, LA strain has emerged as an important marker of atrial function and LV filling pressures [7,8]. A large meta-analysis of healthy individuals reported normal LA reservoir strain (LARS) values around 39%, with a lower limit of normal approximately 23%, and demonstrated an age-related decline in LA strain parameters [25]. Reduced LASR (<18%) has been associated with elevated LV filling pressures, but the cut-offs also depends on LV function [7,8,25]. Although LA strain is influenced by LV systolic deformation and chamber interaction, it integrates information on myocardial properties and filling pressures and has therefore been incorporated into recent diastolic assessment algorithms [7,8].

More recently, diastolic strain rate (SR) has been proposed as a deformation-derived parameter reflecting myocardial relaxation [26,27]. Early diastolic strain rate (SRe) is measured during early LV filling and correlates with LV relaxation and filling pressures [26,27]. The ratio E/SRe has demonstrated prognostic value in the general population [28], as well as in patients with myocardial infarction [29] and aortic stenosis [30], and may outperform E/e′ in risk prediction [28]. Nevertheless, despite strong mechanistic rationale, diastolic SR has not yet been incorporated into routine clinical algorithms, largely due to variability between vendors, limited reference ranges, and insufficient large-scale validation. Current guidelines consider diastolic strain analysis promising but still investigational and not recommended for routine assessment [7,8].

Given that diastolic dysfunction develops early in hypertensive heart disease and may precede global systolic impairment, more sensitive markers of myocardial relaxation are needed. We sought to investigate early diastolic changes in younger hypertensive patients with preserved LV ejection fraction and no significant comorbidities, focusing on the presence of basal septal hypertrophy (BSE). We hypothesized that patients with BSH already exhibit subclinical impairment of diastolic function detectable by conventional echocardiographic parameters and/or deformation-derived indices.

## 2. Materials and Methods

A cross-sectional study was conducted in University Hospital Center Zagreb. Consecutive patients with arterial hypertension who were scheduled for transthoracic examination were included. Consecutive patients were defined as all eligible patients with arterial hypertension referred for transthoracic echocardiography during the study period who met the predefined inclusion criteria. Inclusion criteria were: patients with primary AH, age < 65 years and preserved LV systolic function. Exclusion criteria were: secondary AH, comorbidities (diabetes mellitus, atrial fibrillation, ischemic heart disease, stroke, transitory ischemic attack, and valvular heart disease (more than mild valve stenosis or regurgitation)), bundle branch block and poor acoustic window. The study was approved by Ethics Committee of University Hospital Center Zagreb (8.1-13/115-2, No. 02/21 AG), and all patients signed a written informed consent.

### 2.1. Study Population

Data from patient’s medical history and demographic data were recorded. Comorbidities were excluded according to patient history and medical charts. Ischemic heart disease was excluded by anamnestic data and if necessary by the means of noninvasive (stress test, MSCT) or invasive diagnostic modalities (coronary angiography). Atrial fibrillation and bundle branch block were excluded with electrocardiogram. Blood pressure was measured on both arms according to the recommendations of the European Society of Hypertension, using a calibrated automated upper-arm blood pressure monitor. Measurements were performed after 5 min of quiet sitting in a relaxed position, and the mean value of three consecutive measurements was recorded. Height and weight were self-reported by the participants and body surface area (BSA) was calculated. Sample size estimation was performed based on data from a preliminary pilot study.

### 2.2. Transthoracic Echocardiography

Complete 2D transthoracic echocardiography (TTE) was performed according to standardized protocol [12,31] for patients with AH. GE Vivid 9, General Electric system (GE HealthCare, Chicago, IL, USA) was used. Standardized 2D and Doppler views were recorded and digitally archived, three cycles for each view. For the strain analysis, standard 2D views of the LV were recorded: apical 4-, 2- and 3-chamber views, with minimum temporal resolution of 50 fps and patient holding breath during image acquisition. Collected data were analyzed offline using EchoPac, GE Healthcare, USA. Patients with poor acoustic window were excluded from the study.

**Figure 1:**
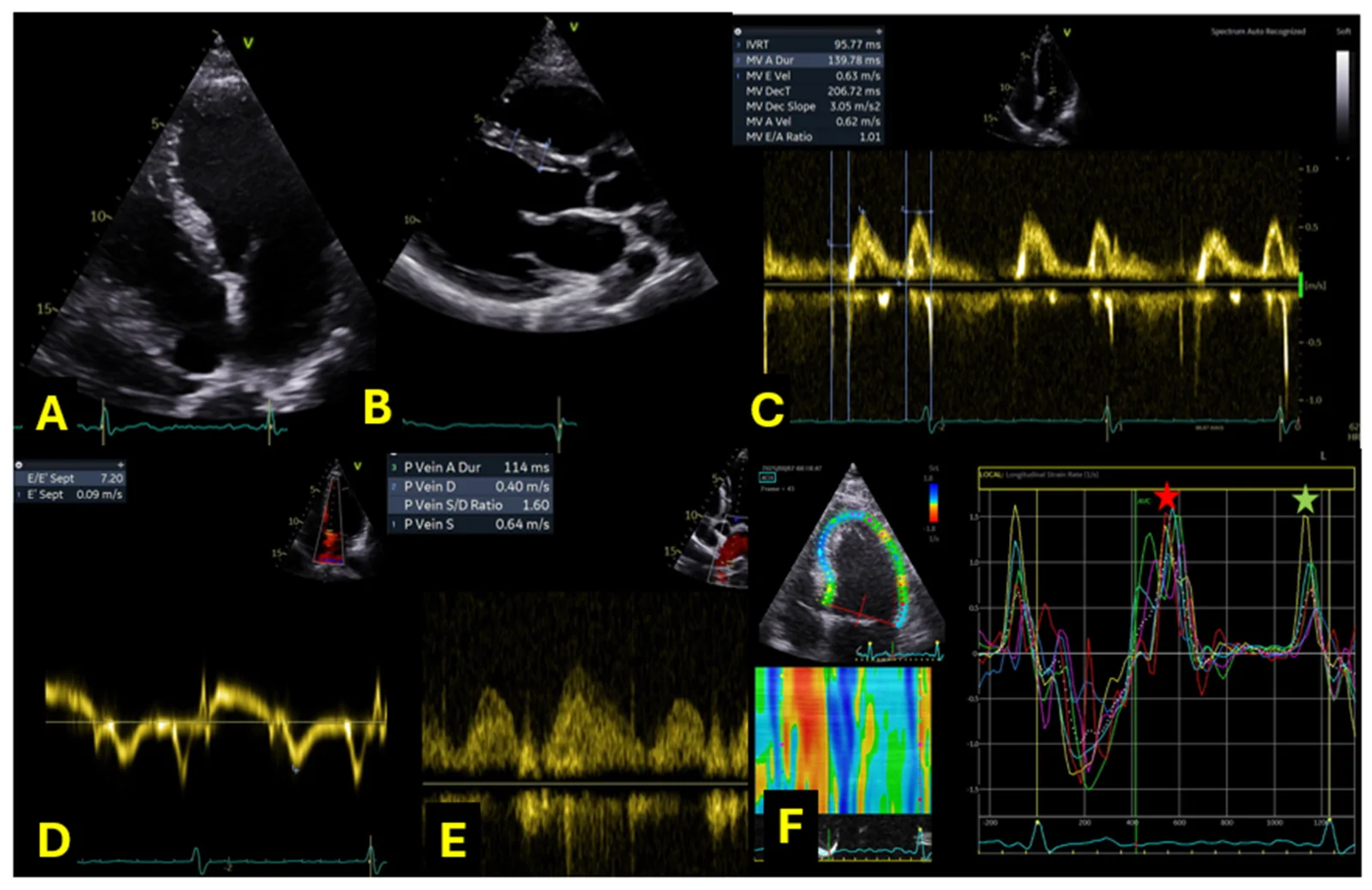
Apical 4-chamber view (**A**) and parasternal long axis view (**B**) showing basal and mid-ventricular septal thickness. Basal iv septum > 2 mm thicker than mid iv septum and/or ratio of basal and mid iv septum thickness > 1.3 define basal septal hypertrophy. Standard measurements of diastolic function on transmitral flow (**C**), mitral annular e′ velocity by tissue Doppler imaging (**D**) and pulmonary venous flow (**E**). Image (**F**) shows speckle tracking measurement of diastolic strain rate during early diastolic filling (SRe, red asterisk) and during atrial contraction (SRa, green asterisk).

Conventional echo data, including LV dimensions, LV mass index (LVMI), left atrial volume (LAV), LV EF and indices of diastolic function, were measured according to the EACVI recommendation papers [7,8,13,31]. Left ventricular EF, end-systolic and and-diastolic volumes were measured using Simpson biplane method. LV mass was measured from parasternal long axis view. Left atrial (LA) volume was measured using biplane method. Values were indexed over body surface area (BSA). Basal septal hypertrophy (BSH) was defined as basal iv septum > 2 mm thicker than mid iv septum and/or ratio between basal septal thickness and mid iv septum thickness > 1.3, according to the previous studies [5,6,21,32,33], as shown in Figure 1. Thickness of the interventricular septum for calculation of RWT and LVMI was measured distally from basal hypertrophy, if present, and thickness of the basal interventricular septum was measured separately at the most thickened basal part (IVSd). Transmitral flow was measured using pulsed wave Doppler on the tips of the mitral leaflets in the 4-chamber view [7,8]. Pulsed tissue Doppler (TDI) was placed on the septal and lateral part of the mitral anulus and peak early diastolic velocity (e′) was measured (e′ sept and e′ lat) [7,8], Figure 1. Ratio E/e′ was calculated.

Patients were divided into two groups according to BSH presence.

### 2.3. Speckle Tracking Echocardiography

Systolic and diastolic indices of LV deformation were measured using speckle tracking method, according to 18-segment model [24,25]. In each of the apical 4-chamber, 2-chamber and APLAX (apical long-axis) views for systolic longitudinal strain (LS) and diastolic strain rate (SR), endocardial border was traced and manually checked. Cardiac cycles were defined as: the onset of systole at the peak of QRS complex automatically set by the machine; aortic valve closure was used as a marker of end-systole and it was set manually by visual tracking in APLAX. The software automatically measured deformation and SR through the whole cardiac cycle. Quality of endocardial border tracing for each segment was checked manually. Global longitudinal strain (GLS) was calculated as an average of 18 segments. Diastolic function was assessed by measuring early diastolic SR (SRe) as a peak velocity during early LV filling, and late diastolic SR (SRa) during atrial contraction, as shown in Figure 1F. Global and segmental values of longitudinal diastolic SRe and SRa were recorded, and values were calculated as an average of 6 segment for each view, and from 18 segments for global measures of diastolic SR for the entire left ventricle. Ratio of E over global SRe (E/SRe) and A over global SRa (A/SRa) as well as ratio of SRe/SRa were calculated.

LA peak systolic/reservoir strain (LARS) was measured in 4- and 2-chamber views; the values were averaged [34,35,36].

Interobserver and intraobserver variability were measured in 10 randomly chosen patients; readers were blinded for previous measurements.

### 2.4. Statistical Analysis

Patient and group characteristics were depicted using descriptive statistics (mean and SD). Patient groups (according to the basal septal hypertophy criteria) were compared using the appropriate statistical test (chi-square, Student *t* test, and Mann–Whitney U test). The intergroup comparison included different demographic, conventional echocardiographic and strain imaging parameters. Finally, correlational and regression analysis were performed in order to establish the relationship among different parameters of diastolic function, left ventricular and left atrial remodeling. Significant regression models were shown graphically. A two-tailed *p* value < 0.05 was considered to be statistically significant for all deployed calculations, while additional one-tailed *p* values were also obtained and provided for comparisons likely to result in one-directional relationships. Analysis was performed using the IBM SPSS Statistics software package (version 26).

## 3. Results

### 3.1. Demographic Characteristics

A total of 138 patients with arterial hypertension (AH) were included (66 men, 47.8%), aged 18–65 years (mean 48.5 ± 11.7 years). Duration of AH ranged from newly diagnosed to 47 years (mean 7.6 ± 9.2 years). None of the patients had significant comorbidities known to influence myocardial deformation parameters.

According to predefined criteria, 74 patients (53.6%) were classified to have basal septal hypertrophy (BSH). Patients with BSH were older (51.4 ± 9.4 vs. 45.2 ± 13.2 years, *p* = 0.006) and had longer duration of AH (9.0 ± 9.7 vs. 6.0 ± 8.5 years, *p* = 0.009). The prevalence of BSH did not differ according to gender (*p* = 0.115).

All patients were on guideline-directed medical therapy; 73% reported regular adherence, with no between-group difference (*p* = 0.051). Elevated home blood pressure (>140/90 mmHg) was reported by 63.8% of patients (*p* = 0.318 between groups). At the time of echocardiography, mean systolic BP was 143.3 ± 19 mmHg and diastolic BP 91.7 ± 13.2 mmHg, without significant differences between groups (systolic *p* = 0.051; diastolic *p* = 0.102).

### 3.2. Conventional Echocardiographic Parameters

All patients had preserved LV ejection fraction (mean 61.4 ± 5.1%). In the whole population overall LV geometry parameters (LV wall thickness outside the BSH region, RWT, LVMI, and LVEDVI) and LAVI were within normal limits, as well as standard diastolic indices, as shown in Table 1.

When comparing standard echocardiographic measurements between groups, there were some significant differences, as shown in Table 1. Patients with BSH had a significant increase in LV wall thickness (measured out of BSH region), RWT and LVMI, i.e., more pronounced incipient concentric remodeling. In the BSH group, there were 31.1% of the patients (n = 23) with RWT > 0.42 (7.8%, n = 5 in no-BSH group, and *p* = 0.001), and 48.6% of the patients (n = 36) with increased LVMI defined according to recommendations [31] (20.6%, n = 13 in the no-BSH group, and *p* = 0.001).

LAVI was mildly increased in the BSH group, and the signs of impaired LV relaxation were notable, as shown in Table 1. Elevated LV filling pressures were identified in 17.4% of patients with BSH compared with 4.7% in those without BSH (*p* = 0.021) [7,8,13].

Age correlated positively with LAVI (r = 0.296, *p* < 0.0001) and with multiple diastolic parameters. With increasing age, E velocity decreased (r = −0.387), A velocity increased (r = 0.333), and E/A ratio declined (r = −0.560) (all *p* < 0.0001). Septal and lateral e′ velocities showed strong negative correlations with age (r = −0.59 and r = −0.548, respectively), while E/e′ showed a weaker positive correlation (r = 0.234, *p* = 0.007).

Duration of AH showed weak positive correlations with LAVI (r = 0.215, *p* = 0.013) and E/e′ (r = 0.256, *p* = 0.003).

Interventricular septal thickness (IVSd) (distal to BSH) correlated significantly with indices of diastolic function: greater IVSd was associated with larger LAVI (r = 0.198, *p* = 0.020), higher E/e′ (r = 0.307, *p* < 0.001), lower E/A ratio (r = −0.199, *p* < 0.001), and reduced septal and lateral e′ velocities (r = −0.369 and r = −0.491, respectively; *p* < 0.001).

### 3.3. Speckle Tracking Strain Analysis

Analysis of systolic strain and diastolic strain rate (SR) was highly feasible. Diastolic SR could be measured in 97.8% of patients in the apical four-chamber view, 97.1% in the two-chamber view, 97.8% in the apical long-axis view, and 97.1% for global diastolic SR.

Interobserver reproducibility for diastolic SR measurements was excellent, with an intraclass correlation coefficient (ICC) of 0.962 for single measures and 0.980 for average measures. The mean relative interobserver difference was 1.28% across all apical views.

#### 3.3.1. Systolic Strain Analysis

Global longitudinal strain (GLS), assessed from three standard apical views, was preserved (−19.7 ± 2.2%) [25]. Global radial strain (GRS) and global circumferential strain (GCS) were measured from basal, mid and apical short-axis views (GRS 43.9 ± 10.8%, GCS −19.5 ± 2.8%). There were no significant differences between BSH and non-BSH groups in any global LV deformation parameter (GLS, GRS, and GCS).

Global LV deformation indices did not correlate with conventional measures of LV diastolic function in this cohort.

#### 3.3.2. Left Atrial Strain Analysis

Peak left atrial reservoir strain (LARS) was assessed in apical four-chamber and two-chamber views [25,35]. In the overall population, four-chamber LARS was 29.77 ± 10.04%, two-chamber LARS 31.93 ± 10.15%, and average LARS 25.51 ± 14.69%. No significant differences were observed according to sex or between BSH and non-BSH groups.

Left ventricular mass index was not significantly associated with average LARS (r = −0.121, *p* = 0.162) or four-chamber LARS (r = −0.149, *p* = 0.121). A modest positive correlation was observed between LVMI and two-chamber LARS (r = 0.323, *p* = 0.013).

**Figure 2:**
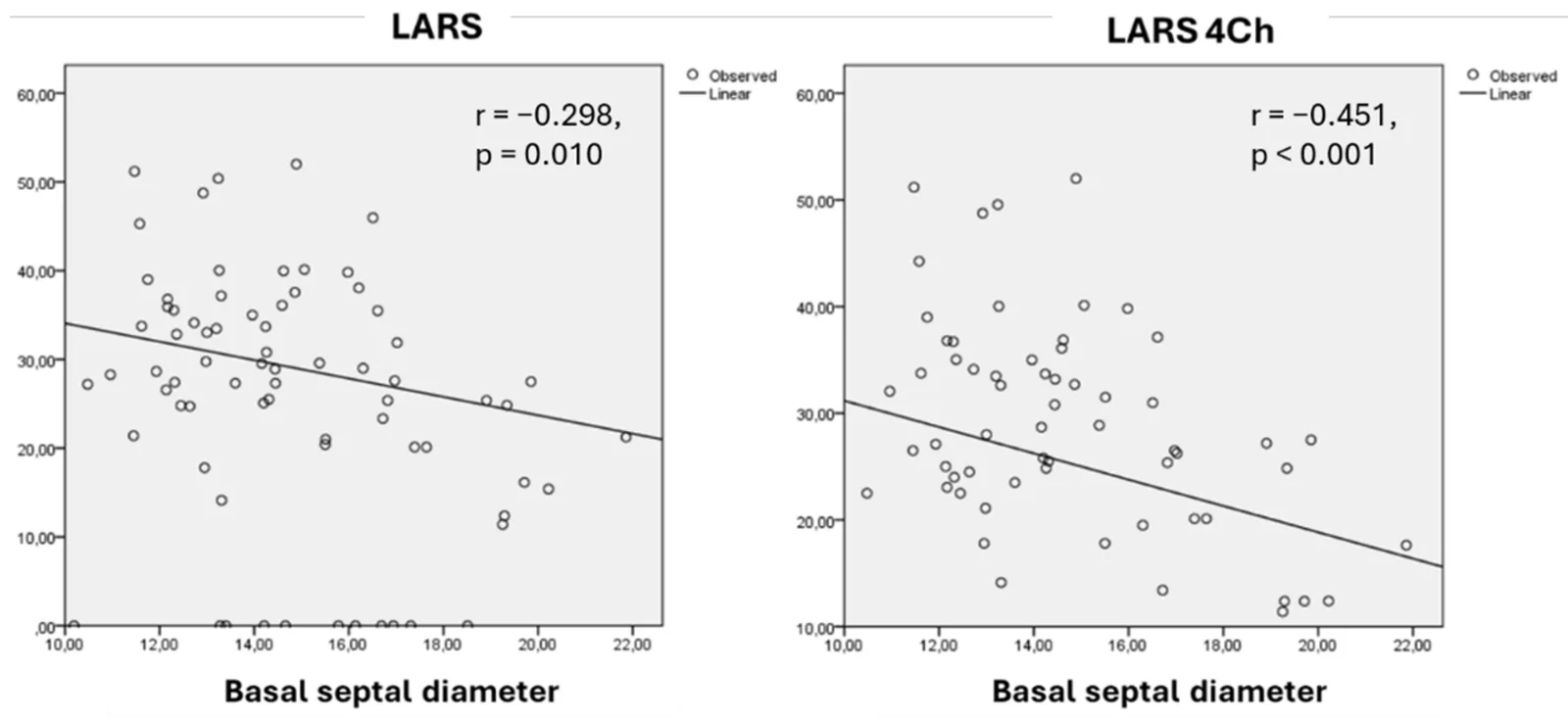
Association between global LARS, 4Ch LARS and basal septal diameter. Increasing basal septal diameter was associated with lower LARS values, especially in 4Ch apical view. The solid line represents the linear regression fit. LARS = left atrial reservoir, 4Ch = apical four-chamber view.

In contrast, basal interventricular septal thickness showed a mild significant inverse correlation with average LARS (r = −0.298, *p* = 0.010). When analyzed by different views, a strong inverse association was observed for four-chamber LARS (r = −0.451, *p* < 0.001), whereas no significant correlation was found for two-chamber LARS (r = −0.157, *p* = 0.339), as shown in Figure 2.

LARS did not demonstrate statistically significant correlations with LA area, LA volume, or LA volume index (all *p* > 0.05).

#### 3.3.3. Diastolic Strain Rate

Diastolic strain rate values during early LV filling (SRe) and atrial contraction (SRa) are shown in Table 2. No significant sex-related differences were observed (SRe 1.18 ± 0.35 in males vs. 1.29 ± 0.36 in females, *p* = 0.11).

However, significant differences were found between BSH and non-BSH group, as shown in Table 2. Patients with BSH demonstrated significantly reduced early diastolic strain rate (SRe) (*p* = 0.02), whereas strain rate during atrial contraction (SRa) did not differ between groups (*p* = 0.44).

**Figure 3:**
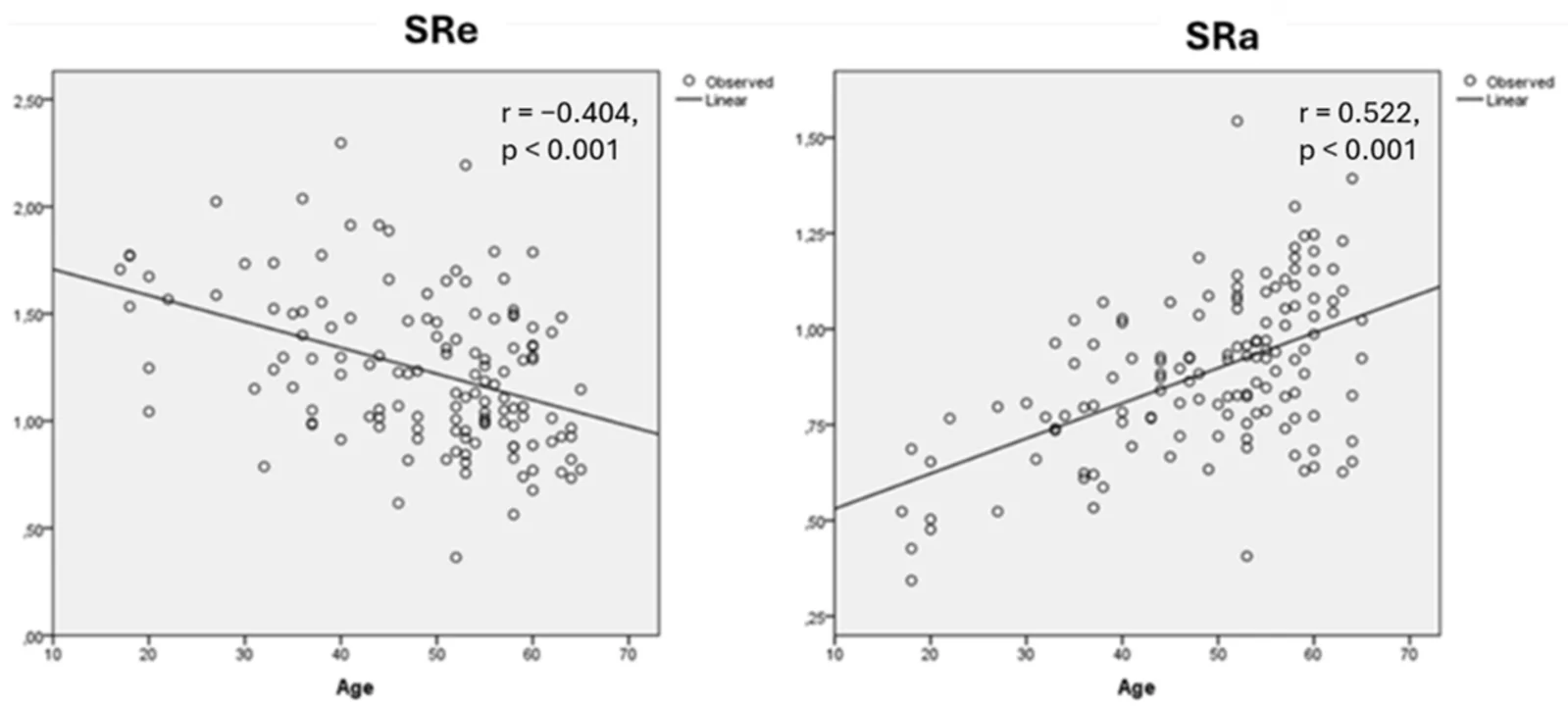
Association between SRe, SRa and age. Increasing age was associated with lower SRe and higher SRa values. The solid line represents the linear regression fit. SRe = early diastolic filling strain rate, SRa = strain rate during atrial contraction.

Age showed strong correlations with diastolic strain rate parameters. Early diastolic strain rate (SRe) correlated negatively with age (r = −0.404, *p* < 0.001), whereas SRa showed a positive correlation (r = 0.522, *p* < 0.001), as shown in Figure 3. Consequently, the SRe/SRa ratio was strongly inversely correlated with age (r = −0.573, *p* < 0.001). In contrast, E/SRe was not significantly associated with age (*p* = 0.26).

Duration of arterial hypertension was weakly but significantly inversely correlated with SRe, both globally (r = −0.235, *p* = 0.007) and across apical four-, two-, and three-chamber views (r ranging from −0.187 to −0.243; all *p* < 0.05). No significant correlations were observed between hypertension duration and SRa.

Conventional diastolic parameters correlated significantly with deformation-derived indices. E/e′ correlated with E/SRe (r = 0.392, *p* < 0.001). E/A ratio showed a strong positive correlation with SRe (r = 0.497, *p* < 0.001) and a strong negative correlation with SRa (r = −0.581, *p* < 0.001).

SRe decreased with increasing LA size, demonstrating weak inverse correlations with LA area (r = −0.225, *p* = 0.009) and LA volume (r = −0.229, *p* = 0.008). Conversely, E/SRe increased with LA enlargement (LA area: r = 0.307, *p* < 0.001; LA volume: r = 0.299, *p* = 0.001).

Increasing basal septal diameter was associated with lower SRe and SRe/SRa values, although the strength of these associations was weak (r = −0.224, *p* < 0.001 and r = −0.135, and *p* = 0.001, respectively). No clinically meaningful association was observed between basal septal diameter and E/SRe (r = 0.055, *p* = 0.050).

## 4. Discussion

### 4.1. Conventional Echocardiographic Parameters

The present study included hypertensive patients younger than 65 years with AH and preserved LV ejection fraction, in whom conventional echocardiographic parameters indicated overall normal LV geometry and systolic and diastolic function [7,8,13,31]. However, despite apparently normal global indices, more than half of the cohort had already developed regional concentric remodeling in the form of basal septal hypertrophy (BSH), reflecting early adaptation to chronic pressure overload (mean AH duration was 7.6 ± 9.2 years).

Duration of arterial hypertension correlated with indices of concentric remodeling and diastolic dysfunction, in line with previous reports [37]. Aging is a well-recognized independent determinant of LV and LA remodeling and relaxation impairment [7,8,13]. Indeed, in our cohort, age correlated with all standard diastolic indices, including E/A ratio, e′ velocities, and E/e′. However, given that our patients were younger than 65 years, the observed alterations cannot be attributed solely to physiological aging. According to current recommendations [7,8], age-related changes in LV filling are expected predominantly in older individuals, whereas our findings suggest that in this younger hypertensive population, regional remodeling occurs due to chronic pressure overload.

Importantly, interventricular septal thickness was significantly associated with markers of impaired relaxation and elevated filling pressures. Greater IVSd correlated with reduced e′ velocities, lower E/A ratio, higher E/e′, and increased LAVI. This pattern indicates that progressive regional septal thickening parallels worsening LV relaxation, rising filling pressures, and secondary LA enlargement. These findings are consistent with the concept that impaired relaxation represents the earliest stage of hypertensive heart disease, preceding overt concentric hypertrophy [4,37,38].

In the overall population, nearly equal E and A velocities (E/A 1.06 ± 0.35) suggest an increased contribution of atrial contraction to LV filling, characteristic of early diastolic dysfunction—impaired relaxation with preserved LV compliance [7,8,39]. Patients with BSH demonstrated more pronounced concentric remodeling and subtle impairment of diastolic function compared with those without BSH, despite preserved global systolic performance. Although e′ velocities remained within guideline-defined normal ranges for age [7,8], they were significantly lower in the BSH group, and a higher proportion of these patients fulfilled criteria for elevated LV filling pressures.

Current recommendations emphasize that when LV systolic function is preserved, assessment of diastolic function requires careful integration of relaxation parameters and filling pressures [7,8]. In early diastolic dysfunction, relaxation is impaired while chamber compliance is still preserved, resulting in reduced early filling velocities but relatively normal filling pressures. Only with progression toward increased stiffness does LV filling becomes restrictive, accompanied by marked elevation of LV diastolic and left atrial pressures [7,8].

### 4.2. Speckle Tracking Strain Analysis

In our study, global deformation parameters remained largely preserved [25] and were not significantly associated with age or conventional diastolic indices, likely reflecting the early stage of hypertensive remodeling in this relatively young cohort. In the older studies, LV hypertrophy and concentric remodeling in patients with AH were correlated to e′ reduction and E/e′ elevation [22,40,41]. Later on, GLS was found to be related to E/A and E/e′ ratio, independently of demographic and other echocardiographic measurements [40,41]. It was reported that GLS independently correlates to E/e′, and that these two parameters can distinguish between normal, athlete and hypertensive heart [22]. In our study, we did not observe such correlation, since global indices of deformation were still near normal. This suggests that systolic longitudinal mechanics are maintained in the early stages of hypertensive remodeling, before the development of overt concentric hypertrophy or more advanced systolic and diastolic dysfunction.

Although longstanding elevation of LV filling pressures is known to result in progressive LA dilation [12,42,43], LAVI in our population remained within normal limits (31.45 ± 10.69 mL/m^2^), but significantly higher and at the upper limit of normal in patients with BSH (*p* = 0.046) [31]. Despite this structural difference, LA reservoir function assessed by LARS did not differ between groups, indicating that atrial mechanics may remain preserved in early remodeling stages since filling pressures were normal at least at rest (since we did not perform exercise stress test).

Importantly, LA strain showed closer relationships with regional septal remodeling than with global LV mass. Basal septal thickness demonstrated a significant inverse association with average and four-chamber LARS, whereas LVMI was not significantly related to average or four-chamber atrial strain. The strong correlation observed in the four-chamber view likely reflects the inclusion of the basal interventricular septum in this imaging plane. In contrast, the absence of correlation in the two-chamber view may be explained by the lack of visualization of the basal septal segment, limiting its ability to capture the mechanical consequences of localized septal hypertrophy. The modest positive correlation between LVMI and two-chamber LARS should therefore be interpreted with caution. This suggests that localized basal septal remodeling, rather than diffuse myocardial hypertrophy, may exert a more direct functional impact on LA mechanics in early hypertensive heart disease [44]. Moreover, LARS is measured in systole, and it occurs in parallel with left ventricular deformation, making it inherently linked to GLS.

Although statistically significant, these correlations were of weak-to-moderate magnitude, indicating that structural remodeling and atrial functional impairment do not progress in strict parallel. This supports the concept that strain-derived parameters may detect subtle functional alterations before overt chamber dilatation becomes evident [25,45].

Furthermore, LARS has been shown to correlate more closely with LV filling pressures than conventional LV systolic parameters and has therefore been incorporated into contemporary British and American echocardiographic recommendations for the assessment of diastolic function and estimation of LV filling pressures [7,8]. In this context, preserved LARS in our relatively young cohort is consistent with the predominantly early stage of hypertensive remodeling and the absence of marked elevation of filling pressures.

Overall, these findings support basal septal hypertrophy as an early, regionally confined marker of hypertensive remodeling with measurable, yet still subtle, functional consequences on left atrial mechanics.

### 4.3. Diastolic Strain Rate

Longitudinal diastolic strain rate analysis was highly feasible and demonstrated excellent interobserver reproducibility.

Diastolic SR represents the velocity of myocardial deformation during relaxation (SRe) and atrial contraction (SRa) and has been proposed as a sensitive marker of early diastolic dysfunction [26,27,39]. In our cohort, early diastolic strain rate (SRe) was significantly reduced in patients with basal septal hypertrophy, while SRa remained preserved. This pattern suggests impaired relaxation with preserved LV compliance—a profile consistent with the earliest stage of hypertensive diastolic dysfunction [7,8].

Importantly, SRe showed strong inverse correlation with age, while SRa increased with aging, resulting in a progressive decline of the SRe/SRa ratio. This age-dependent pattern is consistent with prior observations in healthy populations [46,47]. However, SRe values in our hypertensive cohort were lower than those reported in age-matched healthy individuals [46] and were particularly reduced in the BSH group despite mean age below 65 years. This indicates that pressure-overload-induced regional remodeling, rather than aging alone, contributes to impaired relaxation in this population and the inverse association between hypertension duration and SRe further supports this concept.

The absence of significant changes in SRa and the lack of marked abnormalities in LA strain and E/e′ values reinforce the interpretation that ventricular compliance and filling pressures were still largely maintained.

The ratio E/SRe has been proposed as a novel marker of elevated LV filling pressure [26,27,29]. Its potential advantage over E/e′ lies in the fact that early diastolic strain rate derived from speckle tracking reflects deformation of all myocardial segments rather than only basal annular velocities [29]. Tissue Doppler-derived e′ is limited by angle dependency, load sensitivity, and the inability to distinguish active myocardial relaxation from passive translational motion due to tethering. Furthermore, regional heterogenecity, mitral annular calcification, pericardial disease, prior cardiac surgery, atrial arrhythmias, and right heart pressure overload may complicate interpretation [7,8]. Correlation between E/e′ and left atrial pressure is weaker in patients with preserved LVEF and is influenced by aging and loading conditions [7,8].

Several studies have demonstrated closer correlations between invasively measured LV filling pressures and E/SRe compared with E/e′ in mixed clinical populations [29,30,48,49]. In patients with aortic stenosis, another condition characterized by chronic pressure overload, Dahl et al. showed that E/SRe provided superior prognostic information compared with GLS [29]. Similarly, diastolic deformation-derived indices have shown relevance in mitral annular calcification [30] and myocardial infarction [48].

In our study, no significant differences in E/SRe were observed between BSH and non-BSH groups. This likely reflects the early stage of diastolic dysfunction in our relatively young, otherwise healthy hypertensive population, in whom both transmitral E velocity and SRe were proportionally reduced.

Consistent with prior observations in hypertensive cohorts [39,50], SRe emerged as the most sensitive deformation parameter for detecting subtle functional impairment. With increasing blood pressure, longitudinal systolic and diastolic strain parameters progressively decline, with early diastolic strain rate showing the most distinctive differences [39]. Higher SRa values observed in hypertensive patients likely represent compensatory augmentation of atrial contraction in the setting of preserved compliance and normal end-diastolic pressure [39]. This compensatory mechanism explains why, in our cohort, SRa remained preserved despite reduction in SRe.

Overall, these findings support the concept that early diastolic strain rate is a sensitive marker of incipient hypertensive myocardial dysfunction, preceding overt hypertrophy, significant elevation of filling pressures, or marked impairment of atrial mechanics.

## 5. Study Limitations

This study has several limitations. First, it was conducted at a single center and included a relatively small sample size, which may limit generalizability and reduce statistical power for detailed subgroup analyses. A larger cohort would also allow more robust age-stratified analyses and more precise assessment of the interaction between arterial hypertension, basal septal hypertrophy (BSH), and diastolic parameters. Second, both aging and hypertension influence LV geometry, diastolic indices, and strain parameters, as well as the prevalence of BSH. Although we partially addressed this by analyzing patients younger than 65 years, residual confounding by age cannot be fully excluded. Third, deformation analysis using speckle-tracking echocardiography is dependent on image quality and frame rate, which may affect the accuracy of diastolic strain rate measurements. In addition, current methodology does not allow complete differentiation of early diastolic recoil from atrial contraction-related deformation, which may influence interpretation of late diastolic strain components. Finally, the cross-sectional design precludes assessment of temporal progression and clinical outcomes. Longitudinal follow-up would be necessary to determine whether the observed early mechanical alterations predict future diastolic dysfunction, heart failure, or response to antihypertensive therapy.

## 6. Conclusions

In this study, we demonstrated that diastolic strain rate analysis may detect subtle alterations in myocardial relaxation in younger patients with arterial hypertension.

Although conventional echocardiographic parameters suggested preserved global systolic and diastolic function, the presence of basal septal hypertrophy (BSH) was associated with impaired early diastolic strain rate, indicating subclinical relaxation abnormalities in the absence of overt diastolic dysfunction according to current guidelines.

Our findings are consistent with the concept that abnormalities of myocardial relaxation may occur before global systolic impairment in early-stage hypertensive heart disease. BSH appears to represent an early phenotypic expression of pressure-overload remodeling associated with subclinical impairment of myocardial relaxation, even before significant elevation of filling pressures or diffuse myocardial hypertrophy becomes evident. Diastolic SR in this group of patients provides a reproducible additional measure of early diastolic functional impairment. 

## Figures and Tables

**Table 1:** Standard echocardiographic measurements in patients depending on basal septal hypertrophy (BSH) presence.

Echocardiographic Measurement	All Patients (n = 138)	No BHS Patients (n = 64)	BSH Patients (n = 74)	*p* Value
IVSd (cm) *	1.05 ± 0.23	0.94 ± 0.17	1.13 ± 0.23	<0.001 *
LVIDd (cm)	4.98 ± 0.52	5.01 ± 0.42	4.96 ± 0.59	0.9
LVPWd (cm) *	0.84 ± 0.19	0.74 ± 0.17	0.92 ± 0.18	<0.001 *
IVSs (cm) *	1.54 ± 0.29	1.41 ± 0.26	1.65 ± 0.27	<0.001 *
LVIDs (cm)	2.94 ± 0.55	3.02 ± 0.49	2.88 ± 0.59	0.12
LVPWs (cm) *	1.36 ± 0.25	1.28 ± 0.25	1.42 ± 0.24	0.001 *
RWT *	0.34 ± 0.11	0.3 ± 0.08	0.38 ± 0.11	<0.001 *
LV mass index (g/m^2^) *	99.98± 26.83	89.19 ± 24.59	109.15 ± 25.33	<0.001 *
LVEDV (mL)	82.87 ± 24.04	81.16 ± 23.88	84.29 ± 24.24	0.42
LVEDS (mL)	32.16 ± 10.29	32.18 ± 10.41	32.15 ± 10.25	0.83
LVEDVI (mL/m^2^)	41.02 ± 11.14	40.61 ± 11.01	41.35 ± 11.31	0.7
EF (%) *	61.35 ± 5.07	60.37 ± 4.37	62.18 ± 5.48	0.04 *
LAVI (mL/m^2^) *	31.45 ± 10.69	30.02 ± 11.37	32.68 ± 9.98	0.05 *
E wave (m/s) *	0.75 ± 0.19	0.79 ± 0.2	0.72 ± 0.18	0.05 *
A wave (m/s) *	0.74 ± 0.17	0.72 ± 0.19	0.76 ± 0.14	0.04
Deceleration time (ms)	215.66 ± 61.67	209.09 ± 57.15	221.41 ± 65.23	0.41
IVRT (ms)	82.85 ± 18.87	81.06 ± 19.11	84.43 ± 18.64	0.27
E/A *	1.06 ± 0.35	1.16 ± 0.39	0.96 ± 0.28	0.001 *
e′ septal (m/s) *	0.09 ± 0.02	0.1 ± 0.02	0.08 ± 0.02	0.001 *
e′ lateral (m/s) *	0.13 ± 0.04	0.15 ± 0.04	0.12 ± 0.03	0.001 *
E/e′	8.76 ± 2.57	8.26 ± 2.09	9.22 ± 2.88	0.25
Pulmonary vein flow systole (m/s)	0.59 ± 0.14	0.59 ± 0.17	0.59 ± 0.12	0.61
Pulmonary vein flow diastole (m/s)	0.46 ± 0.14	0.48 ± 0.15	0.45 ± 0.12	0.20
Pulmonary vein atrial systolic reversal wave duration (ms)	99.74 ± 21.13	97.3 ± 22.43	101.8 ± 19.93	0.24

BSH = basal septal hypertrophy, IVSd = interventricular septum diastolic diameter (measured distally from BSH), IVSs = intervent ricular septum systolic diameter (measured distally from BSH), LV = left ventricle, LVEDV = left ventricular end-diastolic volume, LVEDS = left ventricular end-systolic volume, LVEDVI = left ventricular end-diastolic volume index, EF = ejection fraction, LVIDd = left ventricular internal diastolic diameter, LVIDs = left ventricular internal systolic diameter, LVPWd = left ventricular posterior wall diastolic diameter, LVPWs = left ventricular posterior wall systolic diameter, RWT = relative wall thickness. * = statistically significant difference between BSH and no-BSH group.

**Table 2:** Diastolic strain rate measurements.

Diastolic Strain Rate (%/s)	Number of Patients	All Patients	No-BSH Group	BSH Group	*p* Value
4Ch SRe	135	1.23 ± 0.43	1.28 ± 0.42	1.19 ± 0.44	0.16
4Ch SRa	135	0.84 ± 0.23	0.84 ± 0.25	0.84 ± 0.21	0.86
2Ch SRe *	136	1.28 ± 0.41	1.37 ± 0.41	1.2 ± 0.4	0.01 *
2Ch SRa	134	0.92 ± 0.27	0.91 ± 0.28	0.92 ± 0.27	0.47
3Ch SRe *	135	1.22 ± 0.39	1.31 ± 0.42	1.14 ± 0.35	0.02 *
3Ch SRa	135	0.9 ± 0.25	0.86 ± 0.26	0.94 ± 0.24	0.12
Global SRe *	134	1.24 ± 0.36	1.31 ± 0.35	1.17 ± 0.35	0.02 *
Global SRa	134	0.89 ± 0.21	0.87 ± 0.23	0.9 ± 0.18	0.44
E/SRe	133	0.63 ± 0.18	0.62 ± 0.16	0.64 ± 0.19	0.61
A/SRa	133	0.87 ± 0.24	0.86 ± 0.23	0.88 ± 0.24	0.54
SRe/SRa *	134	1.53 ± 0.78	1.69 ± 0.89	1.39 ± 0.63	0.03 *

BSH = basal septal hypertrophy, Ch = chamber, E = rapid filling on pulse wave transmitral flow, A = atrial contraction on pulse wave transmitral flow, SR = strain rate, SRe = early diastolic filling strain rate, SRa = strain rate during atrial contraction, * = statistically significant difference between BSH and no-BSH group.

## Data Availability

The data are not publicly available due to privacy and ethical restrictions concerning the clinical data of individual study participants.
